# Glioblastoma Presenting with Steroid-Induced Pseudoregression of Contrast Enhancement on Magnetic Resonance Imaging

**DOI:** 10.1155/2012/816873

**Published:** 2012-07-11

**Authors:** Marcus D. Mazur, Vinh Nguyen, Daniel W. Fults

**Affiliations:** ^1^Department of Neurosurgery, Clinical Neurosciences Center, University of Utah, Salt Lake City, UT 84132, USA; ^2^Department of Neuroradiology, Clinical Neurosciences Center, University of Utah, Salt Lake City, UT 84132, USA

## Abstract

Corticosteroid-induced reduction in contrast enhancement on radiographic imaging is most commonly associated with lymphoma but has been reported in other entities, including glioma. This finding may represent a diagnostic dilemma. Concern that steroid-induced cytotoxicity obscures histological diagnosis of suspected lymphoma may lead to postponement of a biopsy. If glioma is not considered in the differential diagnosis, reduction in tumor contrast enhancement may be misinterpreted as disease regression rather than a transient radiographic change. We report a case of a patient with an enhancing right temporoparietal mass adjacent to the atrium of the lateral ventricle. After treatment with dexamethasone was started, the mass exhibited marked reduction in contrast enhancement, with symptom improvement. The clinical course suggested lymphoma, and surgery was not performed. Subsequent screening for extra-axial lymphoma was negative. Two weeks later, the patient developed worsening symptoms, and repeat T1-weighted imaging showed interval increase in size and enhancement. The findings suggested a possible diagnosis of malignant glioma. The patient underwent a stereotactic-guided craniotomy for excision of the right temporoparietal mass lesion. Final histological diagnosis was glioblastoma multiforme, World Health Organization grade IV.

## 1. Introduction

Patients who harbor an intracranial mass lesion are frequently treated with corticosteroids to reduce tumor-surrounding edema and symptoms associated with mass effect. Often patients experience substantial improvement in symptoms within 24 hours after corticosteroid administration. Occasionally, the mass lesion may demonstrate a marked decrease in size on contrast-enhanced magnetic resonance (MR) imaging or computed tomography (CT) scans. This feature is most commonly attributed to primary central nervous system lymphoma (PCNSL) [1] but has also been reported in cases of glioma [2–7]. Corticosteroids cause lysis of malignant lymphocytes that may obscure biopsy results, so many physicians recommend avoiding their administration before diagnostic procedures are completed when PCNSL is suspected. Since therapeutic modalities vary widely for PCNSL and glioma, an accurate diagnosis is imperative to ensure appropriate patient care. Consequently, steroid-responsive mass lesions that demonstrate a radiographic change can present a diagnostic challenge. We present a case report of a patient with an intracranial mass lesion who was corticosteroid dependent for symptom management and whose tumor demonstrated marked reduction in contrast enhancement after corticosteroid administration. When the tumor then showed interval increase in size and enhancement, it was eventually diagnosed as an aggressive glioblastoma. We highlight the phenomenon of steroid-induced pseudoregression in gliomas.

## 2. Case Presentation

**Figure 1:**
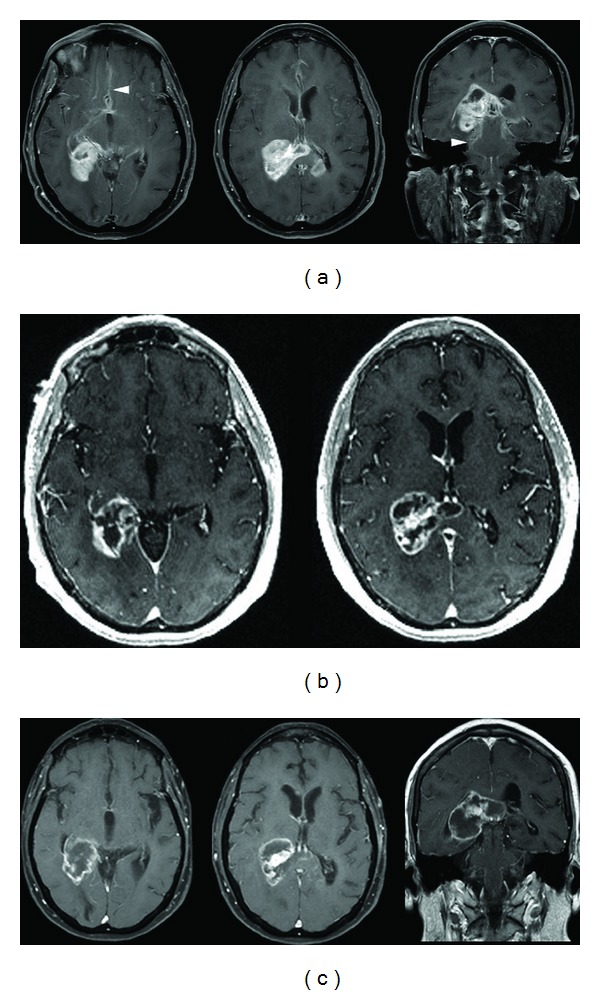
(a) Initial contrast-enhanced axial and coronal T1-weighted fast spin echo (FSE) sequence demonstrating an avidly enhancing temporoparietal mass in a 57-year-old female patient who presented with short-term memory loss, headaches, subtle left-sided weakness, and unsteady gait. There is enlargement of the splenium with nodular enhancement within the contralateral corpus callosum. Extensive areas of subependymal and leptomeningeal enhancement (arrowheads) are present. (b) Contrast-enhanced axial spoiled gradient recalled (SPGR) sequence demonstrating overall decreased enhancement with formation of centrally necrotic areas after 5 days of corticosteroid therapy. The patient's improved functional status and the radiographic regression of the mass suggested a diagnosis of lymphoma. (c) Axial and coronal T1-weighted, contrast-enhanced FSE image obtained two weeks later showing increased nodular enhancement along the inferior and medial margins of the dominant mass and evolution of the necrotic areas. These changes suggested a diagnosis of glioma.

A 57-year-old woman presented with a two-week history of short-term memory loss, headaches, subtle left-sided weakness, and unsteady gait. MR imaging of the brain with a T1-weighted fast spin echo (FSE) sequence showed an enhancing right temporoparietal mass adjacent to the atrium of the lateral ventricle (Figure 1). The referring physician started treatment with 4 mg of dexamethasone given four times daily, which resulted in improvement of the patient's neurological symptoms. A surgical biopsy was initially planned; however, a routine stereotactic MR-imaging scan for intraoperative navigation using a spoiled gradient recalled (SPGR) sequence showed striking reduction in contrast enhancement within the mass (Figure 1(b)). Although there can be changes in the degree of enhancement when comparing a SPGR with an FSE sequence, the reduction in contrast enhancement observed is significantly more than would be expected due to differences in imaging techniques. Because the patient had improved functional status and the tumor demonstrated radiographic change, the clinical course suggested lymphoma, and surgery was not performed. A tapered steroid course was begun, but because of persistent neurological symptoms, the patient was continued on a dose of 2 mg of dexamethasone twice daily. Subsequent screening for extra-axial lymphoma was negative. Two weeks later, the patient developed worsening gait imbalance, and repeat T1-weighted FSE imaging (Figure 1(c)) showed interval increase in size and enhancement of the right temporoparietal mass, a prominent focus of enhancement in the splenium of the corpus callosum, and further leptomeningeal spread. Furthermore, the areas with reduced enhancement on the SPGR image corresponded with areas of evolving necrosis on the follow-up T1-weighted FSE image. These findings suggested a possible diagnosis of malignant glioma. 

**Figure 2:**
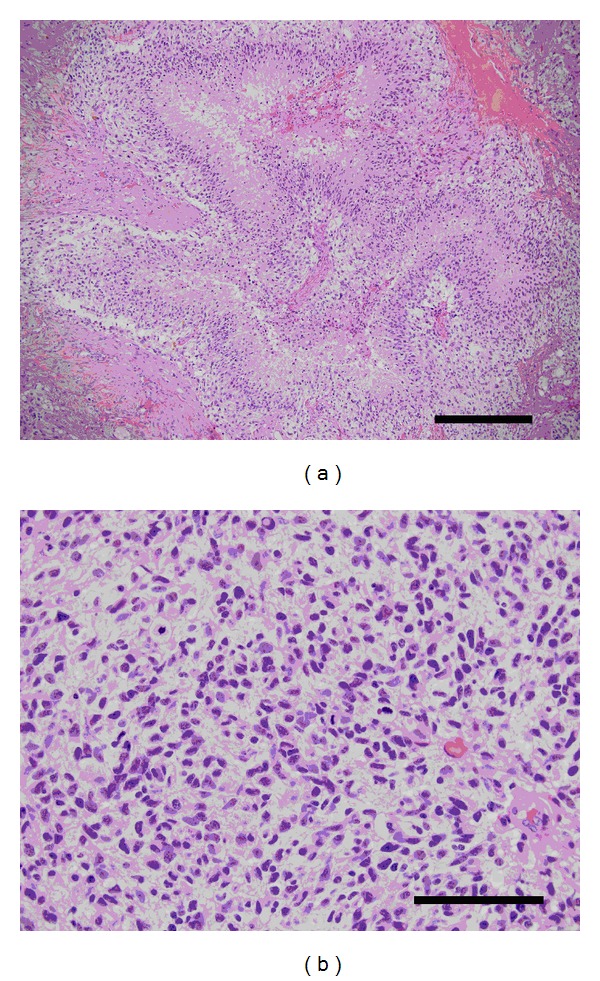
Histological slide from the right parietooccipital biopsy with hematoxylin and eosin staining. (a) Low-magnification micrograph showing serpiginous areas of necrosis with pseudopalisading and vascular proliferation (Scale bar = 0.3 mm). (b) High-magnification micrograph showing hyperchromatic nuclei and frequent mitoses (Scale bar = 0.1 mm). These findings are consistent with a diagnosis of glioblastoma, WHO grade IV.

The patient underwent a stereotactic-guided craniotomy for excision of the right temporoparietal mass lesion. Gross examination of the lesion revealed yellow abnormal tissue of a firm consistency. Biopsy samples were sent as frozen sections to the surgical pathologist. Preliminary results were consistent with malignant glioma. An ultrasonic aspirating device was used to remove the dominant lesion. Tumor tracking from the splenium of the corpus callosum to the contralateral ventricle was not removed. Final histological diagnosis was glioblastoma multiforme, World Health Organization grade IV (Figure 2).

At 1-month followup, the patient reported better cognitive function, resolution of headaches, and improvements in left-sided weakness and gait imbalance. She continued to require 1 mg of dexamethasone daily for symptom management. Neurooncological treatment for glioblastoma continued with a combined regimen of temozolomide and whole-brain radiation therapy. At 2-month followup, she was tolerating her cancer therapy regimen well and remained clinically stable.

## 3. Discussion

Corticosteroid-induced regression of cerebral mass lesions is a characteristic most often associated with PCNSL [1] but is seen less commonly in cases of glioma [2, 4–7], metastatic renal cell carcinoma [1, 8], medulloblastoma [9], multiple sclerosis [1, 10], acute disseminated encephalomyelitis [11], and sarcoidosis [12, 13]. This radiographic finding may impact diagnosis and treatment planning. Corticosteroids can selectively destroy lymphoid cells [14], a property that is used advantageously in chemoradiation treatment protocols for PCNSL but that may obscure a histological diagnosis [15]. Although a complete surgical resection does not improve survival and thus is not usually indicated for PCNSL [16], a tissue biopsy is necessary to confirm the diagnosis. In cases of glioma, conversely, steroids have no significant cytotoxic effects and symptomatic mass lesions often require surgical resection. 

The use of corticosteroids in suspected but unconfirmed cases of PCNSL is controversial. Current clinical practice is to refrain from corticosteroid administration to avoid rendering a tissue biopsy nondiagnostic [17] and delaying appropriate treatment [18]. Cartmill et al. [19] recommended discontinuing the use of steroids for an arbitrary minimum of  5 days to allow the disease to re-establish itself,  followed by repeat imaging shortly before attempting to obtain a biopsy. Some authors, however, have challenged this practice. In a retrospective analysis of biopsy-confirmed PCNSL, Porter et al. [20] noted that corticosteroid treatment did not negatively affect obtaining a histological diagnosis, while Haldorsen et al. [18] did not observe an association between steroid use and biopsy yield. Nevertheless, neither of these studies reported data on lesions that exhibited radiographic change. To our knowledge, aside from individual case reports [15, 21–23], the success rate of obtaining a diagnostic biopsy in lesions that demonstrate change in appearance on contrast-enhanced imaging has not been investigated. 

For glioma, corticosteroid-induced contrast reduction has been observed on both CT- [3] and MR-imaging [6] scans. Watling et al. [6] published a case series of 10 patients who received dexamethasone for recurrent malignant gliomas and underwent baseline MR-imaging scans before treatment followed by weekly scans for 1 month. They reported that 9 of 10 patients had a measurable reduction in contrast enhancement or T2-weighted abnormality with maximal radiographic improvement evident within 2 weeks. Although the precise mode of action remains unclear [24], contrast enhancement of brain tumors depends largely on the permeability of the local blood-brain barrier and is not a direct measure of tumor activity [25]. Clinical trials on modifiers of vascular endothelial growth factor signaling (e.g., bevacizumab and cediranib) in high-grade gliomas revealed early marked reduction in contrast enhancement without significant increase in overall survival [26, 27]. Moreover, although some patients treated with antiangiogenic agents have continued to show reduction in the contrast-enhanced portion of tumor on T1-weighted MR imaging, they have been reported to develop progression of the nonenhancing regions of the tumor on the T2-weighted and fluid-attenuated inversion recovery sequences [28, 29]. This appearance of decreased contrast enhancement without true antitumor activity has been termed “pseudoresponse” [25].

The amount of tumor pseudoregression can be substantial. Cases of gliomas with complete disappearance of contrast enhancement on radiographic imaging after steroid treatment have been reported [2, 4, 5]. Despite the striking reduction in contrast enhancement, corticosteroid-induced imaging change is a transient radiographic phenomenon with little impact on the natural course of glioblastoma. Each of the published case reports that examined patients with glioblastoma after steroid treatment noted tumor reappearance after 1 to 4 weeks from the time of radiographic change [2, 4, 5]. Furthermore, these tumors exhibited many similar characteristics, including multicentricity, involvement of the corpus callosum, and a highly aggressive disease course (Table 1). Despite marked reduction and even complete disappearance of contrast enhancement, 3 of 5 patients included in prior case reports died shortly after such radiographic change was observed [2, 4, 7].

Traditionally, the Macdonald criteria have been used to assess treatment response in gliomas [30]. Measurements of enhancing regions on CT or MR imaging are used to categorize therapeutic impact in terms of amount of radiographic change. Because of the known symptom relief that steroids can confer, the Macdonald criteria downplay clinical improvement and emphasize reduction in contrast appearance on imaging. They rely on the discretion of the physician and investigator to exclude “nontumor-related” causes of clinical or radiographic change and to discern false results from true disease regression. Awareness of tumor pseudoresponse to treatment prompted the development of more up-to-date criteria to evaluate therapeutic response. The Response Assessment in Neuro-Oncology includes specific definitions of the following parameters: radiographic change observed on both T1-weighted, gadolinium-enhanced and T2- or FLAIR-weighted MR imaging; the presence of new lesions; current corticosteroid dose; and the patient's clinical status [31]. 

This case highlights the importance of obtaining a tissue diagnosis in intracranial mass lesions that change in radiographic appearance on gadolinium-enhanced MR imaging. Although most commonly attributed to PCNSL, marked reduction in contrast enhancement may also be seen less commonly in high-grade glioma. Confirmatory testing to ensure an accurate diagnosis is imperative because corticosteroid-induced radiographic change may represent pseudoregression caused by nontumoric effects in glioma or actual disease regression from cytotoxicity in PCNSL.

## Figures and Tables

**Table 1:** Clinical characteristics and outcomes of patients with glioblastoma reported in the literature who demonstrated reduced contrast enhancement after dexamethasone treatment.

Reference	Age	Presentation	Tumor location	Dexamethasone dose	Initial radiographic change	Time to reoccurrence	Treatment	Outcome
[2]	56 M	Right facial palsy, dysphagia, and unsteady gait	Left frontoparietal lobe	2 mg every 8 hours	Disappearance of tumor and contrast enhancement	3 weeks; reappearance, same location	None	Death immediately after tissue biopsy, ~3 weeks after initial radiographic change
[5]	59 M	Headache and confusion	Left parietal lobe	16 mg every 24 hours	Reduction in contrast enhancement	4 weeks; reappearance, same location	Not reported	Not reported
[4]	61 F	Left hemiparesis, paresthesia	Right temporal lobe, right frontal lobe, splenium	4 mg every 6 hours for 2 weeks	Near-complete resolution of all lesions	4 weeks; increased size, new focus	Radiotherapy	Death ~5 months after initial radiographic change
[7]	53 M	Seizure	Right parietal lobe, corpus callosum	4 mg every 6 hours for 3 weeks	Reduced enhancement in right parietal lobe, increased enhancement in corpus callosum	3 weeks	Radiotherapy	Not reported
[7]	75 M	Confusion, short-term memory loss	Right parietal lobe, posterior corpus callosum	4 mg every 6 hours for 3 weeks	Resolution of right parietal lesion, increased enhancement in splenium	3 weeks	None	Death prior to commencing radiotherapy
This paper	57 F	Short-term memory loss, unsteady gait	Right temporoparietal lobe, splenium	4 mg every 6 hours for 5 days	Reduction in contrast enhancement	2 weeks; increased size, new focus, leptomeningeal spread	Chemotherapy with temozolomide, radiotherapy	Clinically stable 2 months after initial radiographic change

## Abbreviations

CT: Computed tomography
FLAIR: Fluid-attenuated inversion recovery
FSE: Fast spin echo
MR: Magnetic resonance
PCNSL: Primary central nervous system lymphoma.
